# 30-days effects of vildagliptin on vascular function, plasma viscosity, inflammation, oxidative stress, and intestinal peptides on drug-naïve women with diabetes and obesity: a randomized head-to-head metformin-controlled study

**DOI:** 10.1186/s13098-019-0466-2

**Published:** 2019-08-23

**Authors:** Alessandra Schiapaccassa, Priscila A. Maranhão, Maria das Graças Coelho de Souza, Diogo G. Panazzolo, José Firmino Nogueira Neto, Eliete Bouskela, Luiz Guilherme Kraemer-Aguiar

**Affiliations:** 1 Postgraduate Program in Clinical and Experimental Physiopathology (FISCLINEX), Faculty of Medical Sciences, State University of Rio de Janeiro, Rio de Janeiro, RJ 20550-013 Brazil; 2 Laboratory of Clinical and Experimental Research on Vascular Biology (BioVasc), Biomedical Center, State University of Rio de Janeiro, Rio de Janeiro, RJ 20550-013 Brazil; 3 Lipids Laboratory (Lablip), Policlínica Piquet Carneiro, State University of Rio de Janeiro, Rio de Janeiro, RJ 20550-003 Brazil; 4 Obesity Unit, Policlínica Piquet Carneiro, Department of Internal Medicine, Faculty of Medical Sciences, State University of Rio de Janeiro, Rio de Janeiro, RJ 20550-030 Brazil

**Keywords:** Diabetes, Obesity, Oral therapies, Endothelium

## Abstract

**Background:**

Obesity is the main risk factor for diabetes and excessive visceral fat triggers low-grade inflammatory process, mediated by activation and release of cytokines and high flow of free fatty acids that contribute to insulin resistance, increased oxidative stress, and impaired endothelial function. Metformin and vildagliptin have known vasculoprotective actions, but the value of these drugs on drug-naïve diabetic patients during 30 days use warrants investigation. Our purpose was to observe their effects on endothelial function, oxidative stress, inflammatory biomarkers, and plasma viscosity.

**Methods:**

38 women with obesity and type 2 diabetes drug-naïve, aged between 19 and 50 years, BMI ≥ 30 kg/m^2^, were recruited and subjected to measurements of endothelial function, nutritive skin microvascular reactivity, plasma viscosity, inflammatory and oxidative stress biomarkers at baseline and randomized 1:1 to ingest metformin (850 mg twice/day) or vildagliptin (50 mg twice/day) during 30 days, and then, re-evaluated.

**Results:**

No differences between groups were noticed at baseline. After treatment, vildagliptin promoted an improvement on endothelial-dependent and -independent vasodilatations, at arteriole level, while metformin resulted in improved nutritive microvascular reactivity, at the capillary level. Intragroup analysis showed that vildagliptin reduced insulin, C-peptide and oxidized LDL, and increased adiponectin and glucagon-like peptide-1 while metformin reduced weight, plasma glucose, total cholesterol, HDL-c, LDL-c, and dipeptidyl peptidase-4 activity, with an unexpected increase on tumor necrosis factor-α. No significant difference in plasma viscosity was noted.

**Conclusions:**

In the vascular beds investigated, both drugs used for only 30 days improved endothelial function, through distinct, and possibly, complementary mechanisms on drug-naïve diabetic women.

*Trial Registration* ClinicalTrials.gov: NCT01827280

## Background

Obesity is a chronic disease, characterized by an increased amount of body fat. Visceral obesity is more related to cardiovascular risk than subcutaneous one since it is currently known that intra-abdominal adipocytes are metabolically more active than ones located at subcutaneous sites. Excessive visceral fat accumulation is not only associated with type 2 diabetes mellitus but also with precocious atherogenic abnormalities [1].

The pathophysiology of excessive visceral fat is related to low-grade inflammation mediated by activation/release of many cytokines, such as interleukin-6 (IL-6) and tumor necrosis factor-alpha (TNF-α). Besides, the increased flow of free fatty acids (FFA) contributes to insulin resistance (IR), also predisposing atherosclerosis and diabetes [2].

Over various periods, patients with obesity maintain glucose homeostasis by hypersecretion of insulin to overcome IR. However, over time and, like obesity and age progress, relative insulin deficiency is observed, triggering glucose intolerance or diabetes diagnosis. Paralleled with this pathophysiology, the occurrence of low levels of incretins, such as glucagon-like peptide-1 (GLP-1) and glucose-dependent insulinotropic polypeptide (GIP) influences insulin secretion [3] negatively.

Diabetes increases macro and microvascular disease risks, but states of glucose intolerance per se would be enough to promote them [4]. Currently, microvascular dysfunction tested on the skin is already present even without altered glucose homeostasis of diabetes’ relatives, being associated with the occurrence of IR [5]. Such finding and other ones reveal that endothelial damage precedes diabetes diagnosis and even the occurrence of glucose intolerance [6–9].

Metformin is the most prescribed drug for T2D treatment. Although this drug has little direct vasomotor effect on large and mid-sized arteries [10], its capacity to improve endothelial function at microcirculatory level has been repeatedly demonstrated, as follows: in type 2 diabetes [11, 12], in persons with impaired glucose tolerance [10] or first-degree relatives of type 2 diabetic patients with metabolic syndrome and normoglycemia [13].

Dipeptidyl peptidase-4 (DPP4) inhibitors, including vildagliptin, are antihyperglycemic agents largely used. Some investigations associate beneficial actions of GLP-1 on endothelial function, and vildagliptin’s incretinomimetic and insulinotropic actions are well-established with known benefits on glucose control [14]. DPP4 inhibitors effects may be associated with the same or even more significant impact on vascular function on diabetic patients.

We aimed to compare the effects of vildagliptin and metformin on vascular reactivity in drug-naïve diabetic women with obesity. We hypothesized that regulatory mechanisms of the possible beneficial vascular actions, especially vildagliptin´s effects, would be related to oxidative stress, inflammation and may also be influenced by incretins.

## Methods

### Study design and participants

This is a short-term randomized head-to-head trial. Selected patients were recruited at our outpatient’s care unit. We exclusively selected female patients since they represent the majority of our population followed on our unit, and additionally, they show a greater adherence in the follow-up. Additionally, gender-specificities on vascular function were reduced by choosing only female patients. This study was conducted according to guidelines set out in the Declaration of Helsinki and approved by the local Research Ethical Committee (COEP: 0.87.3.2012) and registered in the Clinical Trials (NCT01827280).

During the first visit, the research protocol was explained, and the written informed consent was obtained from all participants. Afterward, a medical examination was performed aiming to evaluate inclusion and exclusion criteria, assessment of concomitant drugs in use, physical exam and collection of blood samples. At this visit, patients underwent a 75-g oral anhydrous glucose tolerance test (fasting and 2 h), hepatic enzymes, creatinine, total blood, and leukocyte counts and lipid profile determination after 10 h fast. Diagnosis of diabetes was established if fasting plasma glucose (PG) was ≥ 126 mg/dl or post-load (75 g of glucose anhydrous) PG was ≥ 200 mg/dl [15]. Asymptomatic patients with PG ≥ 126 mg/dl collected another sample to confirm the diagnosis.

The primary inclusion criteria were to have BMI ≥ 30 kg/m^2^ and a diagnosis of diabetes without any previous treatment with any antihyperglycemic agent. Additionally, these patients should be aged between 19 and 50 years old and have an abdominal circumference ≥ 80 cm.

We have excluded women who presented uncontrolled hypertension, or with clinical indication to change antihypertensive dosage during the study. Other exclusion criteria were: pregnant, major illness such as renal or hepatic insufficiency, history of previous myocardial infarction or angina pectoris, postmenopausal women, hematological diseases, triglycerides ≥ 400 mg/dl, being an active smoker, having a significant loss of weight (> 5%) during the last 6 months; or being regular users of aspirin, hormonal contraceptives, anticoagulants and drugs for dyslipidemia.

Two hundred and forty-six women were interviewed and selected for the first visit and screening procedures and, 40 of them were included in the study. The leading cause for exclusion was due to the absence of diabetes, or being diabetic but already on treatment (n = 206). After inclusion, one patient was excluded due to non-compliance (n = 1) and another one due to significant weight loss before the first endothelial test.

The participants were randomized by external selection 1:1 to receive metformin or vildagliptin which was made by an outer member from the study using a random numerical sequence electronically built. To minimize glycemic effects on vascular function, we have designed the experiment to last only 30 days. The total dosage was 1700 mg/day (850 mg/pill) and 100 mg/day (50 mg/pill), respectively for, metformin and vildagliptin; being both ingested twice a day (at lunch and dinner). No patient received both drugs concomitantly. During the first week of treatment, only the dinner pill was taken to minimize gastrointestinal side effects. All participants were informed to keep their usual diet, physical activity and also regular use of other proposed medications in both groups. Except for antihypertensive drugs, which were not changed during the study, no other drug was accepted for use without previous communication. Compliance was tested at days 15 and 30 by counting pills. Criteria for non-compliance was the use of less than 85% of the total dosage/period. The following described measures were assessed before (day 0-baseline) and after (day 30) treatment period.

### Anthropometric, clinical and laboratory measurements

The same trained examiner collected anthropometric measurements: weight (days 0 and 30) using a digital scale (*Filizola*, *São Paulo*, *SP*, *Brazil*) and waist (day 0) at its smallest point with the abdomen relaxed. BMI was defined as weight in kilograms divided by the square of height in meters. Blood pressure was also measured twice in the supine position with a 5-min resting interval between measurements, using an automated apparatus (*Lifewindow LW6000*; *Digicare Biomedical Technology, West Palm Beach, FL*). We also evaluated the percentage of body fat and lean mass through bioimpedance (*Biodynamics 450; Biodynamics Corporation, USA*) at baseline and day 30.

### Endothelial function assessment

We assessed forearm blood flow (BF) by non-invasive venous occlusion plethysmography (*Hokanson*, *EC6*, *D.E*., *Bellevue*, *WA*, *USA*) expressed in ml/min 100/ml of tissue according to the protocol previously described and validated [16].

Before BF measurements, patients rested for 20 min on recumbent position. Venous occlusion plethysmography comprised four phases: first basal flow (b1); reactive hyperemia response after 3 min arterial occlusion (post-occlusive reactive hyperemia response—PORH); second basal flow (b2) and flow 5 min after 0.4 mg sublingual nitroglycerin (NTG) (*Nitrolingual*, *BurnsAdler Pharmaceuticals Inc.*, *Charlotte*, *NC*, *USA*). After PORH, a 15-min interval was given before b2. Blood flows were measured in cycles of 15 s each (10 s venous pressure use followed by 5 s venous pressure release) over 2 min. The mean of the first four measurements in each period was used. Heart rate and BP was continuously measured using a cardiac monitor *(DX 2021, Dixtal Biomedica Ind. Com. Ltda., São Paulo*, *SP*, *Brazil)* adjusted in the dominant arm.

### Skin nutritive microvascular assessment

Nutritive skin microcirculation was tested by videocapillaroscopy performed at the dorsum site of the third finger at resting and after 4 min ischemia according to well-validated methodology [17] by the same observer who was blinded to patient data. Functional capillary density (FCD) before and during post-occlusive reactive hyperemia response (FCD during PORH) that respectively represents the number of capillaries/mm^2^ with blood flow at basal state and after ischemia, using an optical microscope (*DM/LM, Wetzlar, Germany*) with 250× magnification was assessed. This technique was carried out at the baseline and 30 days after treatment.

### Plasma viscosity

Plasma viscosity was evaluated according to previously validated protocol [16]. For this, immediately after blood collection, a tube of 5 ml of blood was centrifuged during 5 min at 1500/*g*. After this, the supernatant was collected, and 0.5 ml was used to test viscosity in plasma (ηp) samples, assessed with a cone-in-plate viscosimeter DV-II + PRO Digital (*Brookfield Engineering Laboratories, Middleboro, MA, USA*) at 230 s^−1^ shear rates and 37 °C. Results were expressed as mPascal × s (mPa s).

### Biomarkers of low-grade inflammation, oxidative stress, and intestinal peptides

IL-6 and endothelin-1 were evaluated by Quantikine^®^ High Sensitivity IL-6 ELISA and Quantikine^®^Endothelin-1 ELISA kits, respectively (*R&D Systems, Minneapolis, MN, USA*). Oxidized LDL was tested by Mercodia ELISA kit (*Mercodia, Uppsala, Sweden*). Urinary levels of isoprostane were evaluated by Bioxytech^®^ Urinary 8-epi-Prostaglandin F_2α_ kit (*Oxis Research*, *Foster City*, *CA*, *USA*). All assays were performed according to manufacturer protocols, and intra and interassay coefficients were < 10%, except for urinary isoprostane analysis which was < 20%. Active GLP-1 were measured by sandwich high-sensitivity ELISA chemiluminescent assay (Merck-Millipore, Billerica, MO, USA).

Multiplexing analysis was used to determine GIP, insulin, C-peptide, ghrelin, leptin, glucagon, pancreatic polypeptide (PP), peptide YY (PYY), adiponectin, resistin, and TNF-α by Magnetic Milliplex^®^ MAP kits (*EMD Millipore*, *Billerica*, *MA*, *USA*). All intra and inter assay precisions were < 10% and < 20%, respectively.

DPP4 activity was tested using glycyl-prolyl-para nitroanilide (*Gly*-*Pro*-*pNA*, *Sigma*-*Aldrich*, *Saint Louis*, *MO*, *USA*) as a chromogenic substrate. At the end of incubation period, the activity of DPP4 in the samples was determined by comparing the optical density of each sample with the optical density derived from a p-nitroaniline standard curve, generated by data analysis software (*KC Junior*, *BioTek*, *Winooski*, *VT*, *USA*). Results were expressed as μM of p-nitroaniline/ml/min. The sensitivity of this method was 0.1 μM/ml/min, and intraassay precision was < 3%.

Ultrasensitive C-reactive protein (CRP) was measured by turbidimetry using high sensitivity latex method (A25 BioSystems^®^, *Biosystems SA, Barcelona, S*pain) and measured. Additionally, the blood cell count was performed by automated hematology counter (XS1000i Sysmex^®^, *Sysmex Corporation*, *Kobe*, *Kansai*, *JPN*). PG, total cholesterol (TC), high-density lipoprotein cholesterol (HDL-c) and triglycerides (TG) were evaluated spectrophotometrically, as follows: glucose oxidase/peroxidase, cholesterol oxidase/peroxidase, direct detergent and glycerol 3-phosphate/peroxidase; using an automated analyzer (A25 BioSystems^®^, *Biosystems SA, Barcelona, Spain*). LDL-c levels were calculated by Friedewald´s equation [18].

### Statistical analysis

We used GraphPad Prism^®^ 5 (GraphPad Software Inc., San Diego, CA, USA) for statistical analysis. Gaussian distribution was checked, and parametric and non-parametric data are expressed as mean ± SD. Comparisons were performed according to the normality of the variables and tested by paired and unpaired *t*-tests or U-test. To correlate them we have used Spearman rank-order test. To establish correlations, we first calculated the delta difference from post minus pre-treatment period (baseline) and then correlated these deltas. We used G*Power 3.1.9.2 (Universit ät Kiel, Germany) to calculate sample size of 17 patients/group. Considering the short follow-up, we expected a dropout rate of 20% and a total of 40 patients (FCD during PORH for group 1 and 2, respectively of 22.8 and 32.6 cap/mm^2^; SD within each of 8.0 cap/mm^2^ according to Buss el al. [17]; *t*-tests; point biserial; two-tailed; effect size of 1.251; α probability error of 0.05; and a power of 0.95). Significant differences were assumed to be present at the level of *P *< 0.05.

## Results

Thirty-eight participants aged 39.4 ± 6.5 years with BMI of 37.2 ± 5.0 kg/m^2^ completed the study. At baseline (Table 1), both groups had the same characteristics on anthropometric, clinical and laboratory variables. Forearm blood flow (FBF) at baseline, during PORH-first curve (C1) and also mean of first four curves (C1–C4), and after sublingual nitroglycerine were the same before treatment period when we compared Vildagliptin to Metformin groups, respectively (3.02 ± 1.81 vs. 2.39 ± 1.56 ml/min/100 ml of tissue, P = 0.34; 10.21 ± 6.55 vs. 8.61 ± 3.59 ml/min/100 ml of tissue, P = 0.63, C1; 5.82 ± 3.06 vs. 4.56 ± 1.69 ml/min/100 ml of tissue, P = 0.19, C1–C4 and 2.55 ± 1.36 vs. 1.97 ± 1.02 ml/min/100 ml of tissue, P = 0.28). These groups also had the same skin nutritive microvascular reactivity before treatment (Table 2).

**Table 1:** Comparison between Vildagliptin and Metformin on clinical, anthropometrical, and laboratory variables (intergroup and intragroup analysis)

Variables	Vildagliptin (baseline)	Vildagliptin (day 30)	Metformin (baseline)	Metformin (day 30)
Age (years)	39.05 ± 5.32	39.05 ± 5.32	39.79 ± 7.7	39.79 ± 7.7
Weight (kg)	94.96 ± 14.03	95.05 ± 14.14	99.51 ± 16.11	98.69 ± 15.70*
BMI (kg/m^2^)	36.03 ± 3.96	35.63 ± 4.10	38.48 ± 6.12	37.29 ± 5.92
Waist circumference (cm)	105.59 ± 10.5	–	106.55 ± 12.05	–
Hip circumference (cm)	116.06 ± 8.99	–	122.68 ± 11.95	–
WHR	0.91 ± 0.05	–	0.88 ± 0.07	–
Systolic BP (mmHg)	125.7 ± 11.91	122.2 ± 12.44	129.1 ± 18.41	122.3 ± 12
Diastolic BP (mmHg)	75.58 ± 13.41	72.26 ± 9.33	75.84 ± 10.37	73.79 ± 8.28
Heart rate (bpm)	75.68 ± 12.68	69.84 ± 10.49	70.05 ± 12.95	73.11 ± 10.89
Fat mass (%)	39.55 ± 3.95	39.32 ± 3.87	41.10 ± 4.25	41.36 ± 3.53
Lean mass (%)	60.45 ± 6.48	60.68 ± 3.87	59.20 ± 6.13	58.64 ± 3.53
Insulin (mIU/l)	1.76 ± 0.90	1.49 ± 0.73**	1.91 ± 1.17	1.57 ± 0.85
Glucose (mg/dl)	200.26 ± 95.07	193.08 ± 109	190.26 ± 74	153.6 ± 61.19***
HbA1c (%)	8.03 ± 1.79	7.37 ± 1.58	7.85 ± 2.03	7.2 ± 2.55
TC (mg/dl)	183.90 ± 36.74	182.1 ± 41.28	198.00 ± 37.8	*187.8 ± 28.23* ^*^
TG (mg/dl)	161.00 ± 79.84	148.4 ± 83.31	141.21 ± 78.66	153.9 ± 72.75
HDL-c (mg/dl)	43.00 ± 8.19	44.11 ± 9.35	46.73 ± 11.28	44.32 ± 9.62*
LDL-c (mg/dl)	108.60 ± 26.28	105.6 ± 23.24	112.90 ± 25.89	112.6 ± 22.57*
VLDL-c (mg/dl)	32.26 ± 15.97	26.44 ± 9.51	28.26 ± 15.73	30.89 ± 14.55
CRP (mg/dl)	1.15 ± 1.38	0.89 ± 0.7	0.92 ± 0.62	0.75 ± 0.46
C-Peptide (ng/ml)	1.74 ± 0.91	1.64 ± 0.74*	1.78 ± 0.91	1.52 ± 0.50
Grelin (pg/ml)	45.58 ± 19.87	47.34 ± 23.28	44.32 ± 26.92	45.28 ± 33.36
GIP (pg/ml)	24.3 ± 14.6	20.48 ± 13.65	20.29 ± 12.28	23.65 ± 17.66
GLP-1 (pM/l)	1.15 ± 1.04	3.62 ± 3.61**	0.98 ± 0.67	*1.47 ± 1.32* ^*&*^
Leptin (pg/ml)	21,880 ± 19,430	21,460 ± 18,257	25,350 ± 13,330	26,250 ± 14,426
Glucagon (pg/ml)	23.47 ± 20.63	29.02 ± 26.4	31.46 ± 53.48	30.82 ± 46.98
Adiponectin (ng/ml)	11,950 ± 11,540	15,107 ± 18,173*	11,590 ± 6151	12,212 ± 6723
Resistin (ng/ml)	34.01 ± 11.45	36.5 ± 13.81	36.06 ± 18.74	35.93 ± 20.01
PP (pg/ml)	30.17 ± 24.19	35.02 ± 34.45	33.26 ± 34.8	29.72 ± 29.43
PYY (pg/ml)	39.24 ± 21.73	36.51 ± 29.42	38.76 ± 36.88	49.51 ± 31.45
IL-6 (pg/ml)	2.117 ± 1.37	2.018 ± 1.213	3.436 ± 4.04	2.69 ± 2.5
LDL_ox_ (U/l)	74.8 ± 31.33	67.31 ± 29.13*	62.13 ± 16.81	62.54 ± 20.04
NEFA (mmol/l)	0.67 ± 0.23	0.64 ± 0.24	0.63 ± 0.25	0.65 ± 0.19
TNF-α (pg/ml)	0.54 ± 0.61	0.42 ± 0.44	0.36 ± 0.90	0.73 ± 1.19**
Endothelin (pg/ml)	1.67 ± 0.55	1.49 ± 0.35	1.62 ± 0.55	1.62 ± 0.58
DPP4_act_ (μM/ml/min)	9.37 ± 3.52	8.05 ± 3.49	9.65 ± 3.73	9.09 ± 3.52*
Urinary isoprostane (pg/Mmol/g/creatinine)	283.2 ± 299.8	233.4 ± 122.3	214.2 ± 123.9	223.9 ± 78.52
Plasma viscosity (30 mPa s)	1.84 ± 0.1	1.88 ± 0.23	1.88 ± 0.14	1.79 ± 0.13
Plasma Viscosity (50 mPa s)	1.84 ± 0.13	1.85 ± 0.21	1.81 ± 0.09	1.78 ± 0.12

Data are expressed as mean ± SD

*BMI* body mass index, *BP* blood pressure, *HbA1c* glycated hemoglobin, *TC* total cholesterol, *TG* triglycerides, *HDLc* high density lipoprotein cholesterol, *LDLc* low density lipoprotein cholesterol, *VLDLc* very low density lipoprotein cholesterol, *GIP* glucose-dependent insulinotropic peptide, *GLP-1* glucagon-like peptide-1, *PP* pancreatic polypeptide, *PYY* peptide YY, *IL-6* interleukin 6, *LDL*_*ox*_ oxidized low density lipoprotein, *NEFA* non esterified fatty acids, *DPP4*_*act*_ dipeptidyl peptidase 4 activity

* Intragroup comparisons: *P < 0.05; **P < 0.01; P < 0.001

^&^Intergroup comparisons: ^&^P < 0.05

**Table 2:** Comparison between Vildagliptin and Metformin on vascular reactivity (intergroup and intragroup analysis)

	Vildagliptin (day 0)	Vildagliptin (day 30)	Metformin (day 0)	Metformin (day 30)
Resting FCD (cap/mm^2^)	24.1 ± 15.65	32 ± 14.69	34.64 ± 20.29	43.61 ± 16.33
FCD during PORH (cap/mm^2^)	20.81 ± 14.72	28.97 ± 13.01	32.40 ± 19.41	*45.75 ± 15.98* ^*&*^

Data are expressed as mean ± SD

*FCD* functional capillary density, *PORH* post-occlusive reactive hyperemia

Intergroup comparisons: ^&^P < 0.05

**Fig. 1:**
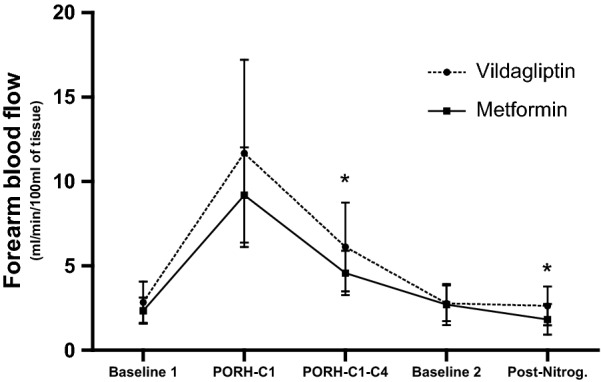
Post-treatment comparisons of forearm blood flow between groups. Data are expressed as mean ± SD. *P < 0.05, for inter-group comparisons. FBF_b1_—Baseline measurement of forearm blood flow, FBF_PORH-C1_—Forearm blood flow during post-occlusive reactive hyperemia (first curve), FBF_PORH-C1–C4_—Forearm blood flow during post-occlusive reactive hyperemia (mean of the first four curves), FBF_b2_—Baseline (post-PORH) measure of forearm blood flow, FBF_NTG_—Forearm blood flow during post-sublingual nitroglycerine

We primarily observed an improvement on endothelial-dependent and -independent vasodilatation on vildagliptin group, while on metformin group we noticed improved nutritive microvascular reactivity at the capillary level. The use of vildagliptin resulted in improvement of vascular reactivity, expressed as augmented responses for endothelial-dependent (during PORH; P = 0.03) and -independent vasodilatation (post-nitroglycerin; P = 0.02) compared to metformin (Fig. 1). Intragroup comparisons did not show any significant differences between pre- and post-treatment periods on both groups.

In respect to nutritive microvascular function at capillary level (Table 2), we noticed that after 30 days of use, metformin group showed higher functional capillary density (FCD) during PORH (i.e., increased capillary recruitment; P = 0.02) compared to vildagliptin. The intragroup analysis did not show any significant difference in FCD at rest (P = 0.49 for vildagliptin and P = 0.55 for metformin) or during PORH (P = 0.21 for vildagliptin and P = 0.10 for metformin, respectively).

After 30 days of treatment, we also observed some changes in some biomarkers (Table 1). GLP-1 levels were significantly higher in the Vildagliptin group compared to Metformin one (P = 0.03). In step further intragroup comparisons were tested, and we noticed that vildagliptin reduced insulin (P = 0.008), C-peptide (P = 0.05), and oxidized LDL (P = 0.02). Additionally, vildagliptin use resulted in increments of adiponectin (P = 0.04) and GLP-1 (P = 0.009) after treatment. In turn, the use of metformin reduced body weight (P = 0.03), fasting PG (P = 0.0005), total cholesterol (P = 0.02), HDL-c (P = 0.03), LDL-c (P = 0.01) and also DPP4 activity (P = 0.03). Of interest, this drug-induced an increase in TNF-α levels (P = 0.008). We did not observe any significant difference in plasma viscosity.

Correlations between delta changes (post-minus pre-treatment) among variables showed some significance, as follows: on Vildagliptin group, FCD during PORH correlated to weight (rho = 0.88, P < 0.01), BMI (rho = 0.88, P < 0.01) and VLDL-c (rho = − 0.73, P < 0.05) while on Metformin group a correlation to PYY (rho = 0.71, P < 0.05) was noticed. In respect to endothelial reactivity, on vildagliptin group a correlation between endothelial-dependent vasodilation and DPP4 activity (rho = 0.46, P < 0.05) was shown while, on metformin group, correlations of this variable to HbA1c (rho = − 0.48, P < 0.05), C-peptide (rho = 0.47, P < 0.05), GIP (rho = 0.54, P < 0.05), IL-6 (rho = 0.65, P < 0.01), adiponectin (rho = − 0.59, P < 0.01), BMI (rho = − 0.55, P < 0.05), plasma viscosity (rho = 0.66, P < 0.05), %body fat (rho = 0.66, P < 0.01) and %lean mass (rho = − 0.66, P < 0.01) were established. Vildagliptin also showed correlation to endothelial-independent vasodilatation and %fat mass (rho = − 0.56, P < 0.05), %lean mass (rho = 0.56, P < 0.05) and heart rate (rho = 0.47, P < 0.05) while metformin showed correlation to plasma viscosity (rho = 0.71, P < 0.05).

## Discussion

Two key features in the pathophysiology of atherothrombosis are closely related to diabetes complications: endothelial dysfunction and low-grade inflammation. The microcirculation is the primary site of the cardiovascular system, responsible for the regulation of tissue perfusion, optimization of oxygen and nutrient delivery and cell/gas excreta and also for the regulation of hydrostatic capillary pressure, preventing fluctuations of intraluminal pressure and, consequently helping to regulate peripheral vascular resistance.

Despite the vast literature about the glycemic effects of these two drugs, scarce comparative data on their vasculoprotective effects was found. A recent multicenter study evaluated whether the combination of vildagliptin and low-dose metformin would improve endothelial function in patients with inadequate glycemic control compared to high dose metformin as monotherapy [19]. In opposite of our study, the authors observed a reduction in BF in both groups after 3 months of follow-up. Possibly, this contradiction could be related to the duration of the diabetes (data not described by the authors) and a worse endothelial function. Probably this group of investigated patients had longer disease duration since 20% of the patients per group had diabetic nephropathy.

In this study, we have employed a method that assessed vascular reactivity at capillary level and also at arteriole one and compared two drugs during short-term use in diabetic patients who had never been subjected to any antihyperglycemic agent. Improvement on endothelial-dependent and -independent vasodilation after vildagliptin use was noticed over metformin use. Endothelial dysfunction appears to be a systemic phenomenon, affecting resistance and conduit vessels not only peripherally but also at coronaries. Endothelial dysfunction at coronary level predicts long-term atherosclerotic disease progression and cardiovascular event rates [20]. Besides, there is also a correlation between forearm vasomotor response (resistance vessels; arterioles) and coronary arteries (conduit vessels) [21].

Consequently, forearm vascular bed can be used as a surrogate marker for systemic endothelial function. On our study, drug-naïve diabetic patients used vildagliptin (100 mg) for only 30 days and improved vascular function. Even though own previous data have shown similar benefits on vascular function with use of metformin on metabolic syndrome patients with normoglycemia during 3 months [13], by using metformin for only 30 days herein, it did not add benefits to vascular reactivity. Although, at the capillary level (by testing it with videocapillaroscopy), this drug resulted in improved capillary recruitment.

One of the aspects to be considered when analyzing our results is the different response of drugs according to vessel caliber. GLP-1 receptor is a G-protein coupled receptor expressed in many tissues, including vessel walls and myocytes [22]. Effects of GLP-1 on blood pressure, heart rate, ischemia/reperfusion injury, coagulability, inflammation, and endothelial function were already observed [23]. Possibly, GLP-1 exerts its effects on nitric oxide production at endothelium [24] and maybe also directly on the smooth muscle cells of the vascular wall [25]. Our findings corroborated it.

On the other hand, metformin is believed to exert its effects through AMP-activated protein kinase, which ultimately increases nitric oxide synthesis and release [26]. Besides, metformin also increases endothelial-derived hyperpolarizing factor-mediated (EDHF-mediated) signaling through processes that improve overall endothelial function, and are likely related to reduced production of cyclooxygenase pathway derivatives [27, 28]. EDHF seems to be more involved in physiological mechanisms on microcirculation. One experimental study tested both drugs on diabetic spontaneously hypertensive rats and noticed that both drugs reduced blood pressure and improved endothelial-dependent relaxation, but while vildagliptin acted mainly through prostaglandins metformin exerted their effects by up-regulating nitric oxide, and more importantly EDHF (through increased plasma sulphide levels) [28]. Therefore, differences observed according to vessel site tested on our study are related not to the period of use but possibly to different control mechanisms of action on distinct tested vascular beds.

In respect to vildagliptin use, the findings described above were glucose-independent, since no change in PG was noticed in this group. GLP-1 bioavailability and the use of DPP4 inhibitors lowers PG in part through increased glucose-mediated insulin secretion and also inhibition of glucagon. On the opposite of our expectations, our study demonstrated lower levels of insulin and c-peptide after 30 days of vildagliptin use. Influences on observed results could initially be associated with a reduction in fasting insulin and C-peptide levels even with higher levels of GLP-1, probably due to lower pancreatic demand found during fasting. Besides, we can assume that the use of vildagliptin over the analyzed period, would reduce pancreatic overload, which ultimately means a reduction in IR. Distinctively from vildagliptin, metformin acts inhibiting gluconeogenesis during fasting, and this feature could have influenced the observed PG changes.

Our study also showed unexpected higher levels of TNF-α after metformin use. TNF-α is increased in obese participants and positively correlated to visceral adiposity and IR. Our sample was initially calculated based on vascular reactivity, and maybe our sample size may have influenced this unexpected result. Blood viscosity is a critical hemorheological factor that regulates blood flow in the microcirculation [29], is considered a cardiovascular risk factor [30]. Our investigation, however, did not reveal significant changes in viscosity.

Our findings also showed correlations between endothelial-dependent and -independent changes with vildagliptin and improvement in body composition, demonstrated by negative correlation with fat mass and positive one with lean mass. These changes, however, were not accompanied by a reduction in DPP4 activity. Despite the good correlation found between vasodilatation in the arteriolar level and body composition, vildagliptin was not able to promote similar correlations in the capillary level.

DPP-4 inhibitors are often added as a complementary choice to background metformin therapy. A recent meta-analysis of three major multinational cardiovascular outcomes trials showed that the addition of a DPP-4 inhibitor reduced cardiovascular events in metformin users [31]. These studies included only individuals with documented cardiovascular disease, which in our research was an exclusion criterion. Therefore the long-term clinical impact of gliptins on cardiovascular risk factors on drug-naïve, newly diagnosed diabetic patients with possibly less advanced comorbid conditions is still inconclusive and should not be inferred by our data.

## Conclusions

In accordance with this premise, we concluded that both drugs, vildagliptin and metformin, used during short-term period, were able to improve vascular function in obese drug-naïve newly diagnosed diabetic women, probably through distinct, and maybe complementary, mechanisms of action on the vascular wall. Vildagliptin acted on arteriole level while metformin acted on nutritive microflow at the capillary level.

In this view, in respect to vascular effects, one class should not replace the other for diabetes treatment. Instead, they are possibly complementary classes of drugs in the search for vascular function improvement and possible protection although long-term protocols are needed to better establish these effects.

## Data Availability

The datasets used and analyzed during the current study are available from the corresponding author on reasonable request. All data generated or examined during this study are included in this published article.
